# Effective factors in people’s preventive behaviors during covid-19 pandemic: a systematic review and meta-synthesis

**DOI:** 10.1186/s12889-022-13621-y

**Published:** 2022-06-18

**Authors:** Hamed Fattahi, Faeze Ghasemi Seproo, Arash Fattahi

**Affiliations:** 1 Center for Primary Health Care Network Management, Deputy for Public Health, Iranian Ministry of Health and Medical Education, Tehran, Iran; 2 Center for Health Human Resources Research and Studies, Iran Ministry of Health and Medical Education, Tehran, Iran; 3 Department of Neurosurgery, Iran University of Medical Sciences, Tehran, Iran

**Keywords:** Covid-19, Pandemic, Preventive Behaviors, Public health strategies, Systematic Review

## Abstract

**Background:**

The overwhelming outbreak of covid-19 has forced governments all over the world to consider different measures to face this challenging situation. A vitally important element to the declining transmission of viruses is changing behaviors based on reliable information. This study was designed and implemented to identify factors affecting the preventive behaviors during the covid-19 pandemic.

**Methods:**

This thematic synthesis was carried out in order to create a set of central themes that summarize all of the issues raised in the articles reviewed in this study. We used PRISMA 2020 guidelines to direct this systematic review and meta synthesis. The process of analyzing data includes three different stages: 1) creating codes; 2) production of descriptive themes; 3) and finally, the creation of analytical themes. The Standards for Reporting Qualitative Research checklist was used to evaluate the articles' quality.

**Results:**

Five central themes emerged from 8 included articles, (1) Social factors (subthemes: environmental context, political leadership, multimedia), (2) Cultural factors (subthemes: national culture, religious culture, the family beliefs, work culture, foreign culture), (3) Economic factors (subthemes: economic situation of the individual, the government supports, infrastructures), (4) Personal factors (subthemes: people experiences, cognitive ability, physical factors, different motivational level, sense of responsibility, risk management, and self-management skills), and (5) Knowledge and Education factors (subthemes: access to information, skill training). Furthermore, SRQR items that were weakly reported were “researcher characteristics and reflexivity”, “Sampling strategy”, “Data collection methods”, “Data analysis”, and “techniques to enhance trustworthiness.

**Conclusion:**

Health policymakers and other public health officials in various countries can use the factors listed to develop appropriate, evidence-based policies. They should investigate behavioral characteristics in their community based on their abilities, and then design and implement appropriate executive actions.

## Background

Since the Second World War, the COVID-19 pandemic has become the most serious disaster worldwide [1]. The overwhelming outbreak of the COVID-19 virus has forced governments all over the world to consider different measures to face this challenging situation [2]. A wide range of measures, including self-isolation, mass quarantine, travel restrictions, lock-down, and the establishment of isolation units and hospitals, were quickly implemented to prevent COVID-19 containment and mitigation around the world [3]. Consequently, not only authorities tried to escalate the number of people who were vaccinated and supported research to find effective treatments, but they also have done many interventions, same as encouraging people to stay at home, mask wearing, avoiding them from social gathering, motivating employees to do their work remotely, and many other activities to restrict virus transportation [4, 5].

On the other hand, it is clear that if people had been extremely concerned about the rapid spread of the COVID-19 outbreak due to its severity and enormous negative effects on public health and society, they would have been more motivated to preventive behaviors [6]. It is a sad truth that the number of people who adhere to prevention and control guidelines has declined in some countries over time. This problem has caused the likelihood of spreading COVID-19, declining the chance of success in implementing governmental policies and plans, wasting worthwhile resources and finally persuading others in society to follow people without commitment to COVID-19 preventive guidelines [7, 8]

In these circumstances, when people perceive a high risk and severity of disease, they take the necessary steps to engage in preventive behaviors. In fact, COVID-19's perceived threat is expected to have a significant positive impact on preventive behaviors because it motivates people to protect themselves against threats [6].

In this regard, a vitally important element to decline transmission of viruses is changing behaviors based on reliable information that is obtained from scientific research or past experiences in any society [9]. For developing effective public health strategies to prevent transmission of covid-19, health policy makers need to be familiar with the virus characteristics, the ways for transmission, the essential preventive behavior to avoid transmission, and finally, the factors that affect public member’s behaviors [10].

Behavioral change in public health is includes a permanent or temporary change in individual attitudes and habits to prevent disease. This change should be followed by five required stages, which are pre-contemplation, thinking, preparation, practice, and keeping [11, 12]. Health policy makers need to understand the components and related factors. Despite that, there is a shortage of reliable evidence for them to develop preventive behavioral strategies to reduce transmission of virus in different countries [13]. Consequently, this study was designed and implemented to identify factors affecting preventive behaviors during the COVID-19 pandemic.

## Methods

### Design

We used PRISMA 2020 guidelines to direct this systematic review and meta synthesis [14] to reach our research goal, which is mentioned in the background. Furthermore, in order to analyze data, we followed Thomas & Harden suggesting method [15]. This process includes three different stages: 1) creating codes; 2) production of descriptive themes; 3) and finally, the creation of analytical themes. In the meantime of this procedure, we used all the suggestions of our academic counselors to assure applicability of the result of this meta-synthesis. Furthermore, the protocol of this study was not registered anywhere.

### Search strategy

In July 2021, we conducted this systematic review and meta-synthesis. Four electronic databases consist of Scopus, Web of Science, Embase, and PubMed were used. We finalized our search strategy by using consultation of our team’s librarian. The search strategy was designed to concentrate on identifying the key words in articles to access more related findings using the keywords: (“people” OR “society” OR “human beings” OR “individuals” OR “persons”) AND (“Coronavirus” OR “COVID-19” OR “Severe Acute Respiratory Syndrome Coronavirus 2” OR “Severe Acute Respiratory Syndrome Coronavirus 2” OR “2019-nCoV” OR “SARS-CoV-2” OR “Pandemics”) AND (“Preventive Behavior” OR “manner*” OR “behav*” OR “demeanor” OR “action” OR “comportment” OR “treatment”).

We used health and multidisciplinary electronic databases, grey literature, manual search in key journals, and finally we did backward and forward tracing of extracted articles’ references. Despite that we searched for all types of articles, the main focus was on qualitative studies. The publication date limitation was from the beginning of the outbreak (2019) to the time search (Sep 2021). Furthermore, we imported the results of searches into Endnote software version 7 (Thomson Reuters, Philadelphia, PA, USA).

### Eligibility criteria

For inclusion criteria in this review study, all papers with a qualitative method that were published in English after 2019 were considered. Moreover, all studies concerning the factors affecting people’s preventive behaviors were included. The exclusion criteria resulted in the non-inclusion of studies, using quantitative methods and not reporting on the preventive behaviors during the COVID-19 pandemic.

### Study selection

For the abstract review, two reviewers initially reviewed the headings and specific articles. Endnote V.7 was used to insert all of the listed headings (Thomson Reuters, Philadelphia, PA, USA). Then, duplicated data was removed. All works on the factors influencing preventive behaviors were included in the abstract review step. Following the restriction of abstracts to those associated with the covid-19 pandemic, a full text review was conducted to classify the studies coping with preventive factors affecting preventive behaviors during the COVID-19 pandemic. In this case, a full review of all full texts was carried out, and specific articles were identified. A cross-search was performed on the references of these papers to identify any additional related articles.

### Quality appraisal

Investigating the quality of the articles was a critical element of this approach for debating the research' results and conclusions, as well as judging the integrity and worth of the data used. We used Standards for Reporting Qualitative Research (SRQR) [16] criteria for qualitative studies to assess the quality of the qualitative publications. No study was excluded from the analysis in our review due to a lack of agreement regarding the function and role of study quality evaluation as part of systematic reviews of qualitative studies [17]. Nonetheless, a small number of high-quality studies appeared to play a considerable role in the synthesis [18].

### Data extraction

The applicable results were extracted and tabulated by re-reading the chosen article, including features of each study, participant demographics, and details of any interesting results. AF was in charge of study selection and data extraction, and HF was in charge of data extraction. Each article was evaluated critically in terms of methodological coherence using criteria defined in the Standards for Reporting Qualitative Research.

### Qualitative synthesis

The method described by Thomas and Harden was used for our analysis [15]. Our investigation began with a thorough reading of each article's headings, abstracts, and texts, followed by re-readings. A single researcher extracted the formal features of the studies (HF). Then, for each study chosen, three researchers (HF, FG, and AF) extracted and analyzed, by coding the text line-by-line, the first-order data (the study results) and the second-order results (authors' discussions and interpretations of the findings), independently. In order to reach saturation point, the process of analyzing data was continued until no new sub-theme was recognized. At the research meetings, we compared and then debated the result of independent analyses. The data was managed and themes developed using the MAXQDA V.18 qualitative analysis software. Thematic analysis enabled the development of themes inductively from study data. The translation phase included comparing and assembling the themes discovered by analyzing each paper in order to retain the key themes, capturing similar ideas in the various articles and then developing overarching ideas about the research question. To achieve a high level of rigor in the data, triangulating both the data sources and the analyses was performed, which included three independent analyses and weekly research meetings to discuss the findings [19].

## Results

### Study selection

**Fig 1.**
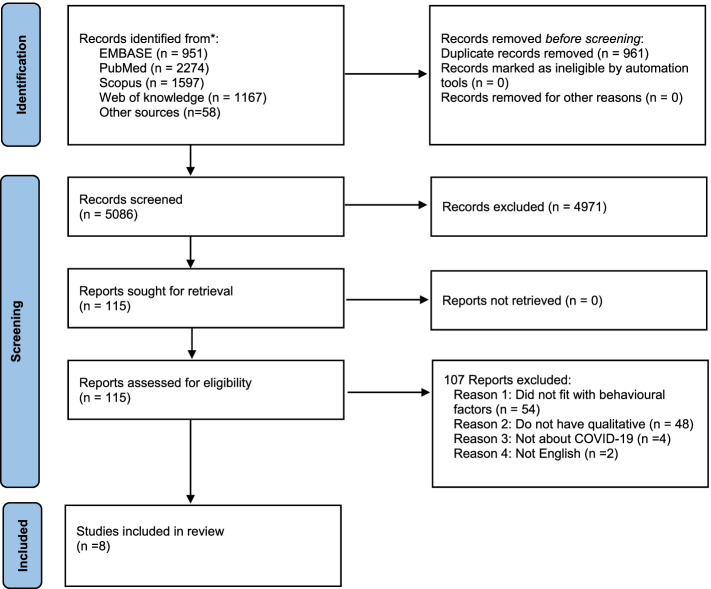
Flow Diagram Showing the Different Phases of Searching for Relevant Publications

First, via searching in the electronic databases, we found 6047 publications related to factors that affected the preventive behaviors during the COVID-19 pandemic. Then, the duplicates (*n* =961) were removed. In the next stage, after reviewing articles by title and abstract, a sum of 4971 studies were excluded by two independent authors, and there remained115 publications for full-text review. After adjusting them with exclusion and inclusion criteria, 8 studies were selected to include in this study [20–27]. Fig. 1 shows the relevant step-by-step exclusion and inclusion of the works in detail.

### Results of the assessment of the methodological quality

Three authors reviewed 8 included studies to appraise them critically. SRQR items that were weakly reported were “researcher characteristics and reflexivity” (none of them), “Sampling strategy”, “Data collection methods”, “Data analysis”, and “techniques to enhance trustworthiness. The poorest score for a study was 6 [21] and the highest was 20 [20] out of 21. Table 1 shows the details of the scoring process. In general, the considered literature provided a rich source of data for the assessment; however, the nature of poorly scoring items was taken into account during the review procedure, which included potential biases in each study.

**Table 1:** SRQR critical appraisal tool results for qualitative studies

**Study**	**Title &Abstract**		**Introduction**		**Methods**											**Results/findings**		**Discussion**			**Others**			**Score**
	Title	Abstract	Problem formation	Purpose or research question	Qualitative approach & research paradigm	Researcher characteristics & reflexivity	Context	Sampling strategy	Ethics pertaining to subjects human	Data collection methods	Collection instruments & tech	Units of study	Data processing	Data analysis	Techniques to enhance trustworthiness	Synthesis & interpretation	Links to empirical data		Integration with prior work, transferability	Limitations		Conflict of interest	Funding	Out of a possible 21
	**S1**	**S2**	**S3**	**S4**	**S5**	**S6**	**S7**	**S8**	**S9**	**S10**	**S11**	**S12**	**S13**	**S14**	**S15**	**S16**	**S17**		**S18**	**S19**		**S20**	**S21**	
**Betsch et al **[21]	X		X		X		X									X			X					6
**Benham et al **[20]	X	X	X	X	X		X	X	X	X	X	X	X	X	X	X	X		X	X		X	X	20
**Bruns et al **[22]	X	X	X		X		X					X				X			X			X	X	10
**Chan et al **[23]	X		X	X	X		X					X				X			X			X	X	10
**Coetzee et al **[24]	X	X	X		X		X									X			X			X	X	9
**Guan et al **[25]	X	X	X	X	X		X									X			X	X				9
**Michie et al **[26]	X	X	X	X	X						X	X	X				X		X			X		11
**Soofi et al **[27]	X	X	X	X	X				X										X			X	X	9

### The characteristics of the included studies

The 8 studies were published in the 2 years from 2020 to 2021. The focus of these studies was on increasing adherence to public health behaviors to reduce COVID-19 transmission. All 8 studies used qualitative designs. Table 2 presents the summary characteristics of the included studies.

**Table 2:** Summary characteristics of the studies (theme reported: I = Social factors, II = Cultural factors, III = Economic factors, IV= Personal factors, V = Knowledge and Education factors)

**Author/year**	**Aim**	**Population**	**Data collection**	**Data analysis**	**Theme reported by authors**
**Betsch et al (2020) **[21]	**To Monitoring behavioral insights related to COVID-19**	**Not exist**	**Not exist**	**Not exist**	**I, III, V**
**Benham et al (2020) **[20]	**To improve health messaging to increase adherence to public health** **behaviors to reduce COVID-19 transmission**	**Populations in Alberta, Canada between August 27** **and September 10, 2020- N=60**	**Focused group discussions**	**Thematic**	**I, II, III, IV, V**
**Bruns et al (2020) **[22]	**To show facts, Cultural Considerations, and Risk of Stigmatization related to covid-19**	**Not exist**	**Not exist**	**Not exist**	**I, II, V**
**Chan et al (2020) **[23]	**To apply an integrated behavior change model of** **health psychology to explain why individuals fail to comply and** **adhere to these behaviors**	**Not exist**	**Not exist**	**Not exist**	**I, II, IV, V**
**Coetzee et al (2020) **[24]	**To find Structural barriers to adhering to health behaviors in the context** **of the COVID-19 crisis**	**Not exist**	**Not exist**	**Not exist**	**I, II, III, IV, V**
**Guan et al (2020) **[25]	**To Understanding the impact of the COVID-19 pandemic on career development**	**Not exist**	**Not exist**	**Not exist**	**II**
**Michie et al (2020) **[26]	**To describe and discuss a systematic method for producing a very rapid** **response (3 days) to a UK government policy question in the context of reducing SARSCoV-** **2 transmission.**	**Not exist**	**Not exist**	**Thematic**	**I, III, IV, V**
**Soofi et al (2020) **[27]	**To understand how insights from behavioral economics can enrich public health policies and interventions in the fight against COVID-19.**	**Not exist**	**Not exist**	**Narrative**	**III, IV, V**

### Thematic analysis results

#### The first theme: Social factors

We seek approval from others, are afraid of being rejected in society, and tend to follow others. What we do and how we think are governed by groups and communities. These are known as social factors that influence Covid-19's related behaviors. Based on the findings of this study, the effective social factors in this regard are classified as follows:

### Environmental context

Environmental factors are the conditions that a person has in a specific environment and situations that can be an encouraging or discouraging factor in creating or updating behaviors in the environment. In this regard, community social approval or disapproval can play an important role in causing an effective change in community members' commitment to preventing the Covid-19 pandemic:

"Not only can this be provided directly by highlighting examples of good practice and providing strong social encouragement and approval in communications; members of the community can be encouraged to provide it to each other. " [26]

While community approval of behaviors strengthens and institutionalizes them in the individual, blame and criticism of individuals can cause weakness and even forgetfulness in performing effective behaviors:

"Discrimination toward, alienation of, and labeling of individuals who wear face masks in public areas or social groups that encourage the ignorance of social distance measures might undermine an individual’s relatedness and subjective norms in the context of COVID-19 prevention" [23]

Not only does approval or disapproval of behavior affect behavior change; the presence of a supportive environment can also lead to people's behavior stabilizing:

"In addition to law enforcement, other social situations and environmental factors are supportive or detrimental to the motivational and social cognition factors affecting COVID-19 prevention" [23]

Another factor influencing people's behavior is their working conditions. This means that if complex programs, such as changing people's behavior in the context of the Covid-19 pandemic, are not implemented at the organizational level and there is insufficient care and support to implement it to change behavior, the process of change in people will face resistance:

"In many low and middle income countries work is often of a physical nature and cannot be done remotely, as may be the case with many white-collar jobs that have been integrated into the digital economy" [24]

One of the most important social factors is the environment in which people must change their behavior. People may face numerous obstacles when attempting to change their behavior in some environments:

"Participants reported experiencing difficulty performing physical distancing in busy, public, indoor settings such as grocery stores, shopping malls, and elevators." [20]

### Political leadership

The importance of political leaders' roles and positions in any society is emphasized. These people can serve as role models for their community.

"Authorities have demonstrated poor role-modeling for face mask use in some settings." [20]

Leaders' attitudes and people's trust in them can influence people in the community to do or not do something:

“I didn’t trust the government’s position in terms of, of the mixed messaging that we receive.” [20]

The use of coercion is also thought to be an appropriate method for changing behavior. However, the effects may be transient and not permanent:

"Experience with UK enforcement legislation such as compulsory seat belt use suggests that, with adequate preparation, rapid change can be achieved where some parts of the population do not initially accept this" [26]

### Multimedia

Using the capabilities and facilities of multimedia tools was one of the tools that drew the attention of many countries following the outbreak of the Covid-19 pandemic. In this regard, many countries have used various applications to inform and guide the public, as well as to implement quarantine laws:

"Some saw contact tracing apps as a good tool for preventing spread, and felt it would be helpful to know if one had been exposed, both for oneself and for others." [20]

In addition, the use of social networks has played a significant role in providing people with the necessary training in a variety of comprehensive fields:

"Public response is closely correlated to the amount of media coverage present for any event. When a health event or crisis is reported on TV, radio, or social media, misinformation can arise and lead to panic, anxiety, and mental health issues." [20]

### The second theme: Cultural factors

Culture is a factor that can have a positive or negative impact on how people behave in society. In the one hand, it makes it easier to achieve change objectives. On the other hand, it can be a barrier to societal change:

"Cultural activities may pose a greater risk for exposure as individuals may not readily avoid such activities. Providers should be aware of those groups and be prepared to screen, treat, and follow-up with infected individuals." [22]

### National culture

National culture of each lands has its own characteristics and features, which are formed by historical, geographical, religious, and ideological elements. In this condition, values are one of the most important aspects of daily life because they reflect the fundamental aspects of human behavior diversity. Differences in various dimensions and areas of collective life (political, social, economic, and so on) are rooted in the selection and preference of one type of value over others.

*"Since cultural values reflect the desirable end states that are worth pursuing, they are likely to influence members' attentiveness to and prioritization of stressors in the appraisal process"* [25]

### Religious culture

Religious and traditional culture are another influential cultural factor in changing behavior. These are known as distinct cultural features that are based on religious and traditional beliefs and values.

"The role of traditional beliefs, including beliefs that inform understandings of traditional healing, is an unexplored area. " [24]

### Family believes

Family beliefs, which can have a significant impact on whether or not individuals adhere to preventative behaviors during epidemics, are another cultural aspect that is heavily influenced by a country's national and religious culture.

*"*Subjective norms (eg, family or friends who are following the COVID-19 preventive strategies say I should do the same)," [23]

### Other kind of culture

Other cultural factors that influence people's behavior include foreign culture and work culture.

#### The third theme: Economic factors

Adherence or non-adherence to measures and behaviors related to epidemic prevention in the population can be significantly related to societal and economic factors.

### Economic situation of individual

The level of income and economic conditions of people in society is one of these factors. As a result, economic problems may reduce individuals' commitment to enforcing laws pertaining to preventive measures in the event of an epidemic:

"Lost wages and the threat of loss of employment may trump concerns about infection or carrying the virus into the home, which may have comparatively less salience for people" [24]

### Government supports

Another factor in combating the COVID-19 pandemic is the use of financial incentives by governments, particularly to engage people with low to moderate financial ability:

“Increasing the current benefit of adherence to social distancing, such as offering small and frequent payments now, can be useful in encouraging people to adhere to COVID-19-preventive behaviors." [27]

Non-monetary incentives can also be effective for this purpose. These incentives will be critical in instilling a sense of worth in people, resulting in a positive attitude toward changing their behavior.

"For example, soaps with toys embedded inside improved hand washing behavior in children. This example is a choice architecture (i.e., nudge) that may nudge children to wash their hands more frequently, so it could be used to increase hand washing during this COVID-19 outbreak." [27]

### Infrastructures

It is clear that countries differ in terms of the amount of access that people in society have to infrastructure. As a result, the presence of flaws or deficiencies in these infrastructures will weaken individuals' adherence to appropriate preventive measures.

"Indeed, in some informal settlements in countries such as South Africa, Nigeria and India, and refugee camps in various parts of the world, the close proximity in which people live makes social distancing and isolation difficult if not impossible" [24]

### The fourth theme: personal factors

Regardless of the importance of external factors, the importance of an individual's personal characteristics cannot be overstated. As a result, in some cases, people's lack of commitment to preventive behaviours should be sought in their personality traits.

"Messaging needs to take account of the different motivational levers and circumstances of different people, informed by the findings from surveys and focus groups" [26]

### People’s experiences

Personal experience in the desired fields is one of the factors that influence people's behavior.

"Many participants who reported that they regularly take the annual flu vaccine felt they would take a COVID-19 vaccine when available." [20]

### Cognitive ability

Ability of everyone to recognize differs, and this can have a significant impact on their behavior:

"Optimism’ as well as ‘Beliefs about consequences’ as important behavioural domains necessary to effect behaviour change" [24]

Knowledge of epidemics such as Quaid-19 is an important factor in the formation of cognition in people.

"Adhering to lockdown optimally will require knowledge as well as skills associated with enacting the desired behaviour." [24]

In that their perception of a behavior's adherence is largely determined by their positive or negative perception of it.

"In particular, when people feel positive about a behavior, they judge its risks as low and benefits as high; when they feel negative about a behavior, they judge its risks as high and benefits as low" [27]

### Physical factors

Individuals' sexual and physical characteristics can also influence their behavior in various situations.

"Among women there are further negative aspects as reports that gender-based violence has increased under lockdown conditions have emerged "[24]

### Different motivational level

Different people are motivated to change their behavior in different ways. As a result, the following considerations should be made:

**"** Some people will be more persuaded by appeals to adhere to government instructions, some by duty to the community, and some to personal risk. Different approaches are needed to take account of this and of the realities of the different lives of people, including their material and social circumstances and their individual needs." [26]

### Other personal factors

Other characteristics of people, such as a sense of responsibility:

"This is important where there is insufficient understanding of, or feelings of responsibility about, people’s role in transmitting the infection to others". [26]

Risk management and self-management skills:

"“. . .every individual has to manage their own risk and what they’re comfortable with. . .it’s a matter of managing yourself and what you’re comfortable with; and your own risk " [20]

### The fifth theme: Knowledge and Education factors

Education is a process that bridges the gap between health information and health behavior by motivating individuals to obtain and apply existing information in order to avoid harmful behaviors and create beneficial behaviors.

### Access to information

Access to reliable and timely information is one of the most important educational factors in changing or creating special behavior during an epidemic such as Covid-19.

"Some participants, particularly in the older age groups, reported that they would be willing to take a vaccine but not right away, instead they would wait for further scientific evidence on vaccine safety and efficacy.” [20]

It is critical to obtain information from reliable sources in this regard:

"communicating through trusted and accessible channels," [21]

Other factors in this area include timely information access:

" Rapid data collection and sharing could support effective interaction between authorities, health workers, journalists, and the public to encourage appropriate behavioral change, to manage the crisis, and to protect the most important asset in a crisis: public trust." [21]

### Skills training

Various factors should be considered in the field of performing skills training for people regarding the creation or change of their behavior in the field of Covid-19. One of these critical issues in education is focusing on providing logical reasons for changing behavior.

"providing more scientific or public health rationale for the importance of these behaviours;" [20]

Additionally, one of the most important issues for people is the development of appropriate and comprehensive guidelines:

" The structure can be improved to help people to understand what actions need to be undertaken, where and when. Guidance needs to be behaviourally specific and structured." [26]

Another critical factor is the method used to deliver the message to the contacts. If this is done correctly in education, it will have a significant impact on people's behavior.

"It seems that health messages intended to encourage people to engage in COVID-19 preventive behaviors (e.g., social distancing) should be framed in terms of gains, such as “If you wash your hands properly/ follow social distancing policy/adhere to the stay-at-home policy, you will increase the chances of yourself and your family having a long, healthy life." [27]

Table 3 illustrates the themes, subthemes, and codes of studies.

**Table 3:** Themes, subthemes and codes of selected studies

**Themes**	**Subthemes**	**Example of codes**	**Sub-themes References**
Social factors	Environmental context	Environments that are supportive of individuals’ basic psychological needs	[20, 23, 24, 26]
	Political leadership	Trust to authorities	[20, 26]
	Multimedia	Using social media and tracing apps	[20–22]
Cultural factors	National culture	Traditional beliefs about health and illness	[22, 24, 25]
	Religious culture	Religious cultures in different areas	[22, 25],
	Family believes	family or friends who are following the COVID-19 preventive strategies	[23, 25]
	Work culture	Different occupational culture	[20, 25]
	Foreign culture	The effect of a foreign culture	[25]
Economic factors	Economic situation of individual	Income, employment rights, and food	[24, 26]
	Government supports	providing free internet access at home	[20, 26, 27]
	Infrastructures	Availability of necessary infrastructures	[21, 24, 26]
Personal factors	People experiences	Effect of experienced side effects with the annual flu shot.	[20, 24]
	Cognitive ability	Risk perceptions influence individual protective behaviors	[20–24, 26, 27]
	Physical factors	Different sex and different level of compliance	[20, 24]
	Different motivational level	Motivating by appeals to adhere to government instructions, some by duty to the community, and some to personal risk	[23, 26]
	Sense of responsibility	Understanding of, or feelings of responsibility about, people’s role	[20, 26]

## Discussion

The reaction of senior managers and policymakers in each country to crises and events determines future situation of the country. Given the prevalence of the Covid-19 virus worldwide, prevention is the most effective method of combating the virus; therefore, all policies, strategies, goals, and programs should rely on existing scientific evidence to maintain and promote public adherence to hygiene precautions are required [28].

Based on the findings of this study, the factors influencing people's preventive behaviors during the Covid-19 crisis were classified into five categories: social factors, cultural factors, economic factors, individual factors, and knowledge and education factors.

Social factors are one of the most influential shaping factors in people's behavior. In fact, these behaviors will be influenced by what people think and feel, what others think and do, and what happens around them that may affect them [29]. As a result, paying attention to social factors and structures, as well as social groups in which people are active, such as occupational, friendship, family, and so on, can play an important role in directing people's behavior toward appropriate or inappropriate behaviors. In this regard, emphasizing social benefits in order to strengthen or weaken certain behaviors is one of the most effective strategies for causing desirable behavior.

Another important factor is the issue of culture, which governs society. The point is that culture, good or bad, does not make sense. On the one hand, community culture can help organizations achieve their objectives. On the other hand, in special and emergency situations, it can be a barrier to change [30]. People from Western Europe, as well as their cultural descendants in North America, are more self-reliant, less conformist, and less submissive, with higher degrees of in-group loyalty [30]. In contrast, some research indicates that areas with a higher prevalence of pathogens have established cultural norms that act as buffers against pathogen transmission, making them more collectivist, because breaking those norms puts the community at danger of infection [31]. Because fundamental and logical cultural change is a time-consuming phenomenon that may even necessitate generational change in a society, there must be a specific strategy in place before developing any program in order to adapt the measures to the cultural dimensions of society.

Factors relating to economic situations make up the third group. Economic conditions in society, families, and individuals, as well as the economic effects of decisions such as quarantine in crisis situations, are factors that behavioral economists should examine in order to provide appropriate policy advice to policymakers in terms of psychological assistance of a much more realistic nature and results [32].

Personality differences are another important factor influencing people's behavior. People engage in certain behaviors for a variety of reasons. According to research, one of the motivators for people to get vaccinated is the desire to protect their friends and family members [33, 34]. In this regard, the motivation strategy can be used to increase people's likelihood of receiving vaccinations. Furthermore, one study found a link between genders and mask use in terms of pain and irritation of the skin, ears, nose, and mouth, among other things [35]. Or another indicated that age differences have a significant impact on COVID-19 protective behaviors, even after mandatory policies were implemented [36]. As a result, to achieve high adherence, the importance of preventive behaviors must be well understood, especially in young people who are less directly threatened by the disease.

One of the first needs of people in crisis situations, such as the occurrence of a pandemic, is to aid in the improvement of knowledge and the implementation of effective training programs both during and before the crisis. In reality, people act at every moment based on the information that is presented to them [34]. According to research, influencing the public to modify its lifestyle through giving information can play a critical role in persuading people to behave in accordance with covid-19 protocols [37]. For example, a person may believe that they are less likely to contract coronavirus based on their past experiences, news they have read, and rumors they have heard. Numerous studies have shown that various media can play an important role in changing and shaping people's behavior in society by providing reliable or untrustworthy information sources [38–40]. Educating and informing members of society in critical situations, on the other hand, is one of the effective factors that can increase people's perceived sensitivity and encourage them to adhere to the desired behaviors. As a result, policymakers should use their capacity for cross-sectoral coordination to provide a roadmap for improving the level of knowledge of people who use their country's existing educational facilities.

### Limitations

The limited number of qualitative studies conducted during the Covid-19 era, which is a qualitative study related to the subject of the research, is one of the limitations in conducting this research. At the time of this research, more valuable studies may have been published. The researchers, on the other hand, attempted to conduct the best possible analysis using the available studies.

## Conclusion

Health policymakers and other public health officials in various countries can use the factors listed to develop appropriate, evidence-based policies. They should investigate their community's behavioral characteristics based on their capabilities, and then design and implement appropriate executive actions. Regardless of the factors influencing preventative behaviors in behavior change, the possibility of successful health-related interventions in emergencies such as the Covid-19 pandemic will be minimized.

## Data Availability

The datasets used and/or analyzed during the current study available from the corresponding author on reasonable request.

## Abbreviations

PRISMA: Preferred Reporting Items for Systematic Reviews and Meta-Analyses
SRQR: Critical appraisal tool results for qualitative studies
